# Netrin-1 Prevents Rat Primary Cortical Neurons from Apoptosis via the DCC/ERK Pathway

**DOI:** 10.3389/fncel.2017.00387

**Published:** 2017-12-13

**Authors:** Jianhao Chen, Houwei Du, Yixian Zhang, Hongbin Chen, Mouwei Zheng, Peiqiang Lin, Quan Lan, Qilin Yuan, Yongxing Lai, Xiaodong Pan, Ronghua Chen, Nan Liu

**Affiliations:** ^1^Department of Neurology, Fujian Medical University Union Hospital, Fuzhou, China; ^2^Institute of Cerebral Vascular Disease of Fujian Province, Fuzhou, China; ^3^Department of Rehabilitation, Fujian Medical University Union Hospital, Fuzhou, China; ^4^Key Laboratory of Brain Aging and Neurodegenerative Diseases, Fujian Key Laboratory of Molecular Neurology, Fujian Medical University, Fuzhou, China

**Keywords:** Netrin-1, primary cortical neurons, DNA damage, DCC, ERK, OGD

## Abstract

In the nervous system, Netrin-1 serves as a neural guide, mediating the neuronal development. However, it remains blurred whether Netrin-1 can protect neurons from apoptosis induced by cerebral stroke. In the current study, the cultured rat primary cortical neurons were transfected with Netrin-1-encoding lentivirus before the oxygen-glucose-deprivation (OGD) treatment. Cell death and apoptosis were evaluated by lactate dehydrogenase (LDH) release and flow cytometry. We found that Netrin-1 attenuated OGD-induced cell death and neuronal apoptosis at 24 h after OGD treatment, and that the overexpression of Netrin-1 activated the ERK signaling pathway. These effects were partly abolished by blocking its receptor deleted in colorectal cancer (DCC) or U0126, an inhibitor of the ERK signaling pathway. Netrin-1 overexpression in neurons elevated the expression of DCC, on mRNA level and protein level. Netrin-1 also reduced DNA damage. Taken together, our findings suggest that Netrin-1 attenuates cell death and neuronal apoptosis via the DCC/ERK signaling pathway in the cultured primary cortical neurons after OGD injury, which may involve the mediation of DNA damage in the neurons.

## Introduction

Cerebral stroke often features the occlusion or hemorrhage of cerebral vessels and results in physical dysfunction or even death of the organisms. It is responsible for almost half of the acute neurological disorders. Cerebral ischemia induces a deprivation of oxygen, which can endanger neuronal survival or damage the neurons by triggering overlapped cell signaling pathways. Consequently, necrosis and apoptosis, which contribute to the neuronal death, will follow suit (Nakka et al., 2008). Therefore, protecting neurons from the ischemic damage remains a priority in treating cerebral stroke conditions.

In the nervous system, Netrin-1, as a guide in neuronal migration, attracts axons towards the middle of spinal cord, which is mediated by deleted in colorectal cancer (DCC; Forcet et al., 2002), and promotes neurogenesis in the striatal primordium (Hamasaki et al., 2001). It also plays a chemorepulsive role in axons of trochlear, trigeminal and facial nerves, which is mediated by Unc5-related proteins (Yung et al., 2015). Moreover, Netrin-1 plays a key role in angiogenesis after stroke. Studies document that Netrin-1 stimulates the proliferation and migration of human cerebral endothelial cells (HCECs) and human aortic smooth muscle cells (HASMCs; Fan et al., 2008; Lu et al., 2012) and that Netrin-1 overexpression in mouse brain promotes angiogenesis and long-term neurological recovery after transient focal ischemia (Fan et al., 2008; Lu et al., 2012). Recent research into tumors reveals that Netrin-1 is also involved in the inhibition of tumor cell apoptosis (Thiebault et al., 2003; Paradisi and Mehlen, 2010). In the research of Mazelin et al. (2004), Netrin-1 promoted colorectal tumorigenesis by inhibiting the cellular apoptosis. But its receptor, DCC can induce apoptosis conditionally, in which DCC acts as a dependent receptor to induce apoptosis. When DCC engaged with Netrin-1, the pro-apoptotic effect was abolished. Netrin-1 also acts as a survival factor in other research (Furne et al., 2008; Delloye-Bourgeois et al., 2009b; Harter et al., 2014). However, little literature is available to illuminate its anti-apoptotic activity in neurons that are subject to oxygen-glucose-deprivation (OGD) condition, which mimics the clinical cerebral ischemic situations.

In tumor research, Netrin-1 inhibits the ERK signaling pathway by combining with UNC5B in pancreatic ductal adenocarcinoma, indicating that the pathway is closely related to the mediation of Netrin-1 (An et al., 2016). In different pathologic conditions, the ERK signaling pathway is involved in apoptosis (Chen et al., 2015; Tan et al., 2016; Lewinska et al., 2017; Weng et al., 2017). In the research of Ma et al. (2010), one of Netrin-1 receptors, DCC, activates the ERK signaling pathway. We speculated that Netrin-1 overexpression could protect cortical neurons from apoptosis by activating the ERK signaling pathway via the mediation of DCC expression. As the ERK signaling pathway has also been documented to affect DNA damage (Dai et al., 2008; Gao et al., 2013; Lee et al., 2013; Tian et al., 2013), we wondered whether Netrin-1 may protect primary cortical neurons after OGD through reducing DNA damage.

In the current study, cultured rat primary cortical neurons were transfected with Netrin-1 encoding lentivirus and subsequently subjected to OGD treatment to mimic an ischemic condition. We found that Netrin-1 attenuated OGD-induced cell death and neuronal apoptosis and that the overexpression of Netrin-1 activated the ERK signaling pathway. These effects were partly abolished by blocking its receptor DCC or U0126, an inhibitor of the ERK signaling pathway. Additionally, Netrin-1 reduced DNA damage via the DCC/ERK pathway. Altogether, these findings suggest that Netrin-1 can improve the outcome of cerebral ischemia.

## Materials and Methods

### Animals

Pregnant Sprague-Dawley rats were purchased from Animal Center of Fujian Medical University (Fuzhou, China). Animals were fed and housed in standard conditions. Animals were euthanized with isoflurane which contained 3% induction, 1.5% maintenance in 30% O_2_ and 70% N_2_O. The protocols followed the guidelines of National Institute of Health (NIH Publications No. 80-23, revised in 1996). The experiments were approved by Institutional Animal Care and Use Committee of Fujian Medical University.

### Primary Cortical Neuron Cultures

Primary cortical neurons were cultured as described previously (Chen et al., 2016). Briefly, samples were prepared from embryonal brains (aged 16–18 days) of Sprague-Dawley rats. The cell suspensions were seeded on 6-well cell culture plates or coverslips (24 mm × 24 mm), with poly-L-lysine coated and cultured in neurobasal medium containing 2% B27, 0.5 mM of glutamine and 50 U/ml of penicillin/streptomycin. The harvested cells were cultured at 37°C with 5% CO_2_. The neurobasal medium was refreshed in 8 h and then half of the medium was refreshed every 2 days.

### Lentivirus Transfection

The cultured neurons were transfected with lentivirus which overexpressed Netrin-1 (the Lv-Nnt-1 group) or vector (the vector group) at an multiplicity of infection (MOI) of 1 according to the manufacturer’s instruction (Genechem, Shanghai, China). Eight hours after transfection, the cultured neurons were processed for various studies. Cells transfected with lentivirus contained the gene that encodes green fluorescent protein (GFP). The transfection efficacy of lentivirus was 96%.

### Oxygen-Glucose Deprivation (OGD)

The OGD model was established as described previously with modifications (Chen et al., 2016). In the vector group and Lv-Nnt-1 group, 7 days after plating, the cultured primary cortical neurons were incubated with glucose-free DMEM and further incubated in an anaerobic chamber, which contained 5% CO_2_ and 95% N_2_, at 37°C for 120 min. Then the cultured neurons were switched back to the pre-OGD culture condition. The control group (CTL group) was not subject to OGD exposure.

### Drug Treatment

For neurons that were transfected with the lentivirus that encodes Netrin-1, U0126 (CST, USA), a specific inhibitor of the Raf/MAPK/ERK signaling pathway, was added to inhibit the phosphorylation of ERK, with a final concentration of 20 nmol/L (the Lv-Nnt-1 + U0126 group). The drug was added to the medium after OGD. DCC blocking antibody (5 μg/mL; Santa Cruz, CA, USA) was applied to the cultured neurons of the Lv-Nnt-1 group right before and after OGD to antagonize the effect of DCC (the Lv-Nnt-1 + anti-DCC group).

### Quantitative PCR (qPCR)

Total RNA was isolated from the cultured neurons with TRIzol according to the instructions. RNA was reversely transcripted to cDNA. The SYBR Green I qPCR kit (Takara, Japan) was used for quantitative PCR following the manufacturer’s protocol. qPCR was detected in triplicate. The gene expression of each sample was normalized to the expression of GAPDH. The qPCR condition was incubated at 95°C for 10 min before 45 cycles of 95°C for 10 s, 63°C for 5 s and 72°C for 15 s. Melting curve was analyzed at 65°C, with the temperature increasing at a rate of 1°C every 10 s to 95°C. The primers used for the research were as follows: Netrin-1, 5′-CCGTGGTGACCAGAGTTTGT-3′ and 5′-ATCACCAGGCTGCTCTTGTC-3′; UNC5B, 5′-CGACCCTAAAAGCCGCCCC-3′ and 5′-GGGATCTTGTCGGCAGAGTCC-3′; DCC, 5′-ACATCCGACGTTCGGCTTT-3′ and 5′-TGATTTTCCCATTGGCTTCC-3′; GAPDH, 5′-CCCTTCATTGACCTCAACTA-3′ and 5′-CCAAAGTTGTCATGGATGAC-3′. GAPDH was used as the reference gene and the expression of each targeted gene was analyzed by 2^−ΔΔCT^ method. Moreover, the control group was used as a calibrator sample and was set as 1× expression of each targeted gene. Three independent experiments were performed.

### Western Blot

The expressions of Netrin-1, ERK, p-ERK and DCC from the cultured neurons were analyzed by western blot after the drug treatment. Briefly, cell extracts were obtained from primary cortical neurons using RIPA lysis buffer containing 1 mM PMSF and Protease and phosphatase inhibitor cocktail (Beyotime, China). The supernatant was centrifuged at 15,000× *g* at 4°C for 10 min. The protein concentration was determined by BCA kit (Thermo, USA). Equal amount of total protein (20 μg) from every sample was separated by 10% or 12% SDS-PAGE and subsequently transferred to PVDF membranes (Millipore, USA). The membranes were blocked with the blocking solution (Beyotime, China) for 1 h and then incubated with primary antibodies at 4°C overnight: rabbit anti-DCC antibody (1:200, Santa Cruz, CA, USA), rabbit anti-ERK antibody, rabbit anti-pERK antibody (1:400, Cell Signaling Technology, USA, respectively); Rabbit anti-Netrin-1 antibody and rabbit anti-GAPDH antibody (1:1000, Abcam, UK, respectively). After three washes with PBST, the membranes were incubated with goat anti-rabbit IgG-HRP secondary antibody (1:8000, Abcam, UK) at room temperature for 2 h and the signals of membranes were detected with ECL reagent kits (Beyotime, China). Band intensities were analyzed with the ImageJ software (1.46r). The relative expression levels of proteins were normalized to the appropriate internal control. Four separate experiments were conducted.

### Cell Viability Assessment

Lactate dehydrogenase (LDH) leakage, as an indicator to the integrity of cell membrane, was used to assess the viability of neurons. It is based on LDH transformation of pyruvate to lactate in the presence of reduced nicotinamide adenine dinucleotide (NADH). The transformation of NADH to NAD is accompanied by a decrease in absorbance at 340 nm and the change in absorbance correlates with the LDH activity in the medium. LDH leakage was measured and calculated according to manufacturer’s protocol of LDH assay kit (Beyotime, China). Four separate experiments were conducted.

### Flow Cytometry

ANNEXIN V-APC/7-AAD Staining was employed to detect the apoptosis of neurons. Flow cytometry was performed as described previously with minor modifications (Lin et al., 2015). In brief, neurons were cultured in flasks (25 mm^2^), following the protocol described above. Twenty-four hours after OGD, AnnexinV-APC/7-AAD staining was performed in accordance with the manufacturer’s instructions. The neurons were resuspended and washed three times with PBS (4°C). Then the neurons were resuspended with 200 μL incubation buffer and then 5 μl of Annexin-V labeling reagent and 10 μl of 7-AAD were added into the medium. The neurons were incubated at room temperature in the dark for 15 min. At least 1× 104 cells were recorded in each sample and the result was analyzed by flow cytometry (Beckton Dickinson, USA). The experiment was repeated three times.

### Immunofluorescence Staining

Twenty-four hours after OGD, imunofluorescence staining was performed to evaluate the DCC expression. Neurons were washed with PBS for three times and then fixed in 4% paraformaldehyde (pH 7.4) for 15 min. Cells were incubated at 4°C overnight with rabbit anti-DCC antibody (1:20, Abcam, UK). After three washes with PBS, they were further incubated with corresponding secondary antibody, Cy3 donkey anti-rabbit IgG (1:400, Jackson Immunoresearch, USA) at room temperature for 2 h. The nuclei were stained with DAPI (5 μg/ml; Beyotime, USA). Glass slides were viewed under a ZEISS LSM 780 confocal microscope (Carl Zeiss, Germany), and the OD was quantified with ImageJ software as described previously. All trials were repeated three times.

### Comet Assay

The comet assay was used to assess the DNA damage of neurons. After the application of coverslips, the slides were allowed to gel at 4°C for about 60 min. The slides were immersed in cold lysing solution at 4°C for at least 1 h, and refrigerated overnight before the alkali treatment. They underwent electrophoresis for 20 min at 1.6 V/cm and 300 mA and then neutralization. The dried slides were subsequently stained using ethidium bromide (20 lg/ml) after appropriate fixing for 10 min. The whole procedure was performed in dim light to minimize artifact. DNA damage was analyzed at a magnification of 200× under a fluorescence microscope (Nicon Eclips E6600, Japan). A total of 50 cells were examined per slide. The tail length and tail DNA% were used to measure the double-strand breaks. Fifty cells were analyzed each time with the Comet Assay Software Project.

### Statistical Analysis

Data were expressed as Mean ± SEM and analyzed by SPSS 20.0 (IBM, USA). Three independent experiments were conducted for all measurements. Statistical significance among groups was determined by one way analysis of variance (ANOVA) followed by Student-Newman-Keuls multiple comparisons test when equal variances were assumed. When equal variances were not assumed, Dunnett’s T3 was applied. Data are presented as mean ± SEM. The significance of mean differences between two groups was calculated by unpaired two-tailed Student’s *t*-tests. The results of western blot, cell death rate and comet assay were calculated by Mann-Whitney U-test, *P* values less than 0.05 (two-sided) were considered as statistically significant.

## Results

### The Expression of Netrin-1 and p-ERK Was Assessed after OGD Across Different Time Points

**Figure 1:**
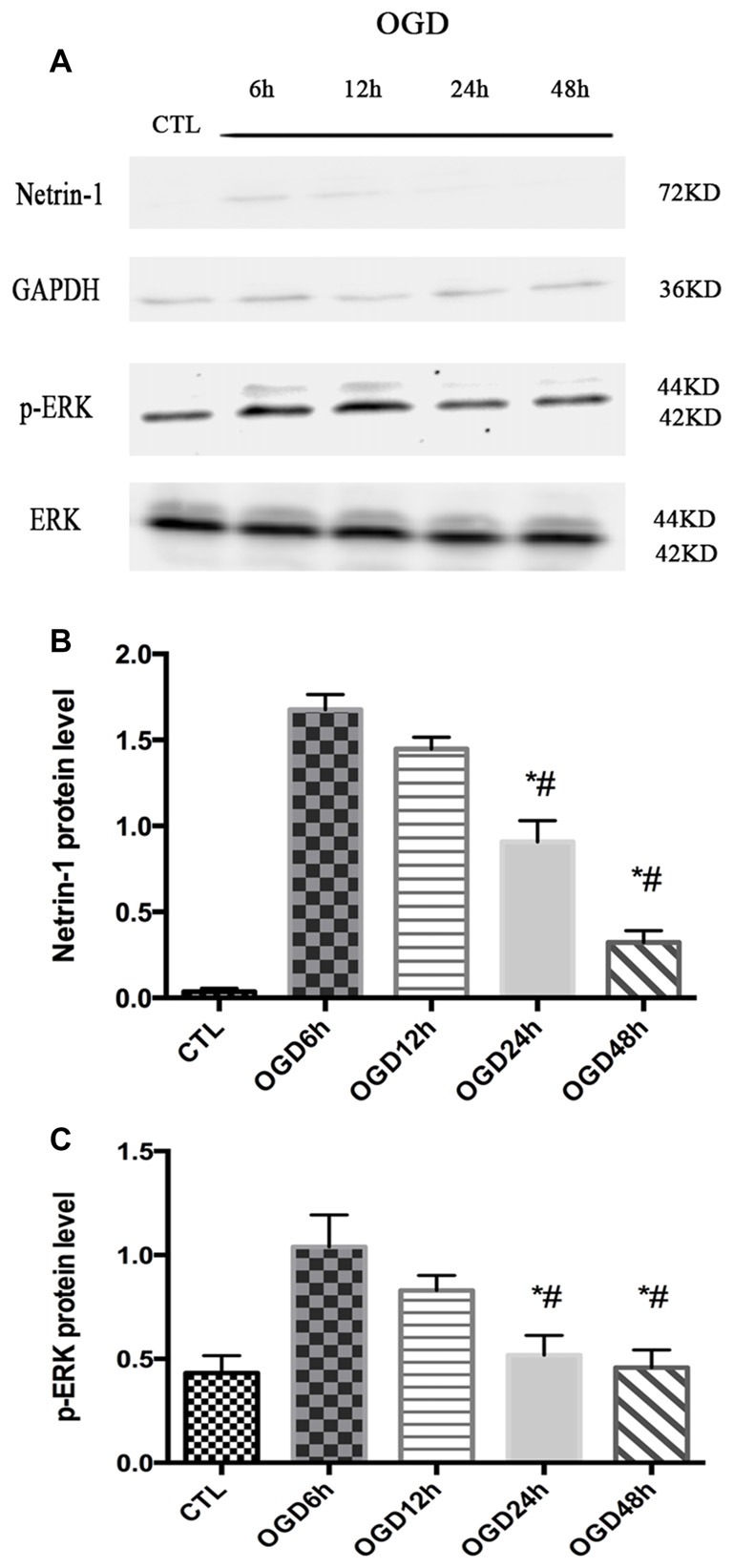
The expression of Netrin-1 and p-ERK in oxygen-glucose-deprivation (OGD) condition across different time points. **(A)** The images of the result of western blot. **(B)** The analysis of result of western blot for Netrin-1. Data are presented as mean ± SEM. *p < 0.05, as compared with the OGD 6 h group; ^#^*p* < 0.05, as compared with the OGD 12 h group; Mann-Whitney U-test; *n* = 4. **(C)** The analysis of result of western blot for p-ERK. Data are presented as mean ± SEM. *p < 0.05, as compared with the OGD 6 h group; ^#^*p* < 0.05, as compared with the OGD 12 h group; Mann-Whitney U-test; *n* = 4.

The expression of Netrin-1 and p-ERK was assessed after OGD across different time points (6 h, 12 h, 24 h and 48 h) by western blot. The expression of Netrin-1 and p-ERK both increased at 6 h and 12 h compared with control group (Netrin-1 expression: 1.04 ± 0.08 vs. 0.04 ± 0.01, *p* < 0.05 and 1.45 ± 0.03 vs. 0.04 ± 0.01, *p* < 0.05; p-ERK expression: 1.06 ± 0.11 vs. 0.43 ± 0.04, *p* < 0.05 and 0.83 ± 0.04 vs. 0.43 ± 0.04, *p* < 0.05), but decreased at 24 h (Figure 1).

### Primary Cortical Neurons Were Transfected with Lv-Netrin-1

**Figure 2:**
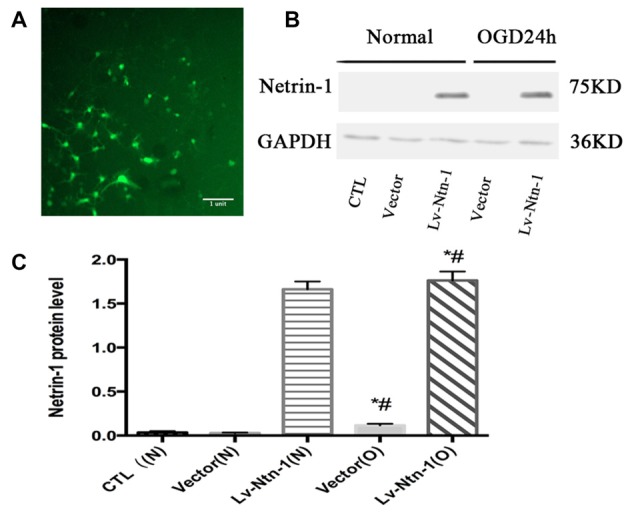
The overexpressed Netrin-1 lentivirus transfected into primary cortical neurons. **(A)** The lentivirus containing gene green fluorescent protein (GFP) was transfected into primary cortical neurons. **(B)** The image of the result of western blot. **(C)** The analysis of result of western blot for Netrin-1. Data are presented as mean ± SEM. *p < 0.05, as compared with the vector (N) group; ^#^*p* < 0.05, as compared with the Vector (O) group; Mann-Whitney U-test; *n* = 4.

Western blot analysis demonstrated that the protein level of Netrin-1 was overexpressed in the Lv-Ntn-1 group when compared with that of the vector group in normal condition and OGD condition (normal condition: 1.66 ± 0.04 vs. 0.033 ± 0.01, *p* < 0.05; OGD condition: 1.76 ± 0.05 vs. 0.12 ± 0.01, *p* < 0.05). In normal condition, the Netrin-1 expression in the vector group was not significantly increased when compared with the control group (0.03 ± 0.003 vs. 0.03 ± 0.01, *p* = 0.486). These results indicate that the transfection of Netrin-1-encoding lentivirus was effective in the primary cortical neurons (Figure 2).

### Netrin-1 Protects Primary Cortical Neurons from OGD-Induced Cell Death and Apoptosis

**Figure 3:**
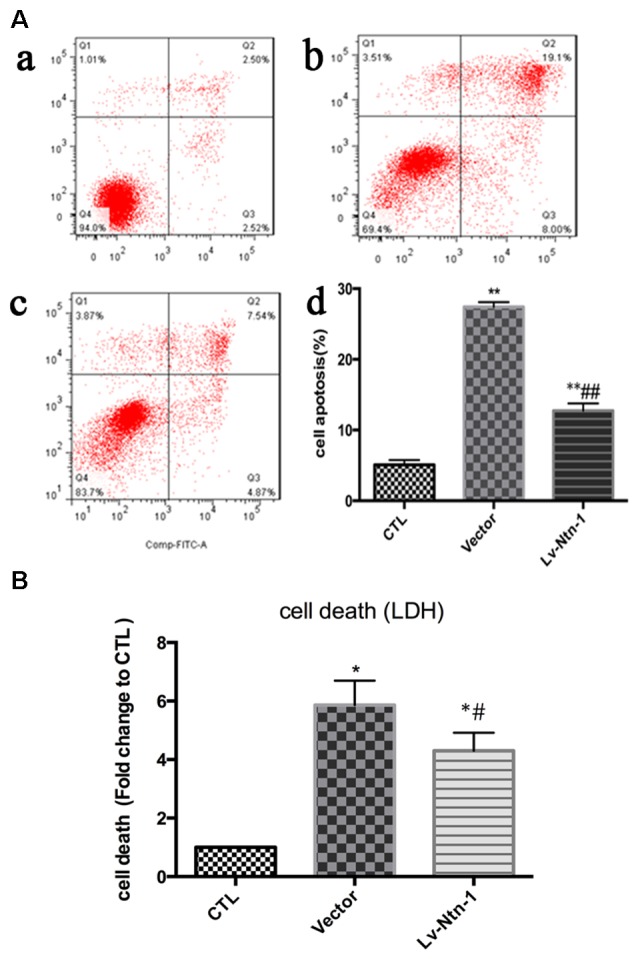
OGD-induced cell death and apoptosis attenuated by Netrin-1 expression. **(A)** Twenty-four hours after OGD, cell death and apoptosis were detected by lactate dehydrogenase (LDH) release and flow cytometry. The signals detected in Q2 and Q4 quadrants represented the apoptotic neurons. **(a)** Control group; **(b)** Vector group; **(c)** Lv-Ntn-1 group. **(d)** The analysis of the result of flow cytometry. Data are presented as mean ± SEM. *p < 0.05, as compared with the control group; ^#^*p* < 0.05, as compared with the vector group; **p < 0.01, as compared with the control group; ^##^*p* < 0.01, as compared with the vector group; One-way analysis of variance (ANOVA; *F*_0.05(2,6)_ = 587.439); *p* < 0.001; *n* = 3. **(B)** Cell death rates of each group, which were measured by LDH release. Data are presented as mean ± SEM. *p < 0.05, as compared with the control group; ^#^*p* < 0.05, as compared with the vector group; Mann-Whitney U-test; *n* = 4.

After the transfection of lentivirus in the cortical neural cells, the effect of Netrin-1 on OGD-induced cell death and apoptosis were further investigated. The death rates of cells after OGD were detected by LDH release, and cell apoptosis was measured by Annexin V-APC/7-AAD. The death rate of primary cortical neurons in the Lv-Nnt-1 group significantly decreased when compared with that of the vector group (5.87 ± 0.42 vs. 4.30 ± 0.31, *p* < 0.05). The cell apoptosis of the Lv-Nnt-1 group also markedly decreased when in comparison with the vector group (12.73 ± 0.60% vs. 27.48 ± 0.36%, *p* < 0.01; Figure 3).

### Netrin-1 Protects Primary Cortical Neurons from OGD Injury via the ERK Pathway

**Figure 4:**
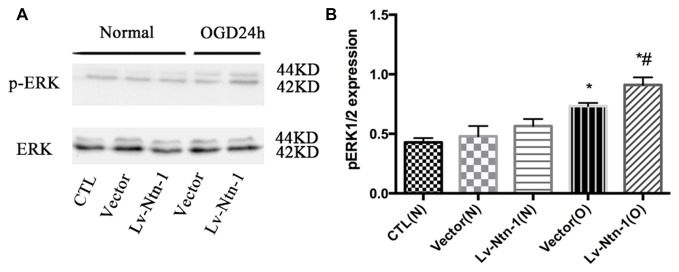
Netrin-1 activated the ERK signaling pathway of primary cortical neurons after OGD. **(A)** The image of the result of western blot. **(B)** The analysis of result of western blot for p-ERK. Data are presented as mean ± SEM. *p < 0.05, as compared with the vector (N) group; ^#^*p* < 0.05, as compared with the Lv-Ntn-1 (N) group; Mann-Whitney U-test; *n* = 4.

**Figure 5:**
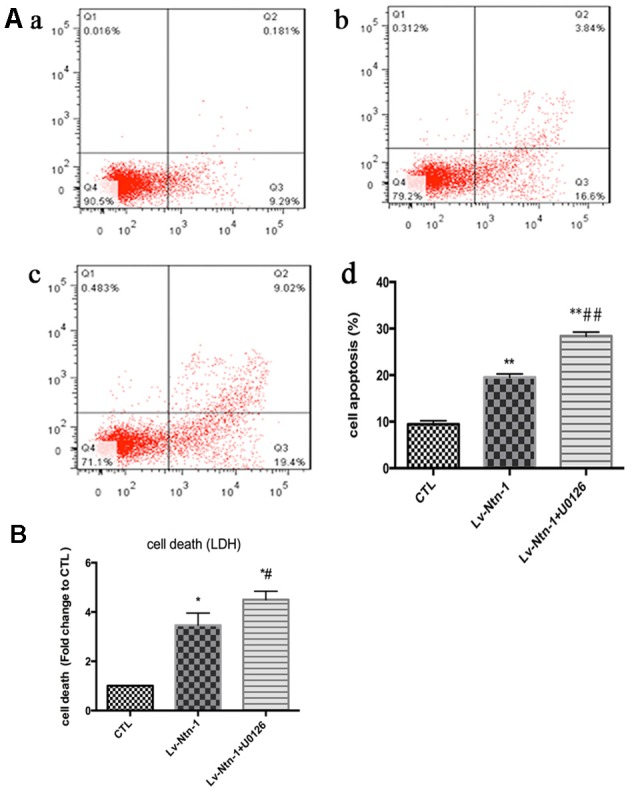
Netrin-1-induced anti-apoptosis was abolished by the inhibitor of ERK signaling pathway, U0126. **(A)** The image of apoptosis detection by flow cytometry. **(a)** The control group; **(b)** The Lv-Ntn-1 group; **(c)** The Lv-Ntn-1 + U0126 group. **(d)** The analysis of the flow cytometry. Data are presented as mean ± SEM. *p < 0.05, as compared with the control group; ^#^*p* < 0.05, as compared with the Lv-Ntn-1 group; **p < 0.01, as compared with the CTL group. ^##^*p* < 0.01, as compared with the Lv-Ntn-1 + U0126 group; One-way ANOVA (*F*_0.05(2,6)_ = 284.425); *p* < 0.001; *n* = 3. **(B)** The analysis of cell death rates by LDH release. Data are presented as mean ± SEM. *p < 0.05, as compared with the control group; ^#^*p* < 0.05, as compared with the Lv-Ntn-1 group; Mann-Whitney U-test; *n* = 4.

As reported in existing literature, the activity of ERK pathway was involved in the neuronal apoptosis. Therefore, we speculated whether Netrin-1 affected the activity of ERK. Lentivirus was transfected into different groups. In the Lv-Nnt-1 group, the phosphorylation of ERK was increased in OGD condition but not in normal condition, which indicated that Netrin-1 induced ERK phosphorylation in the primary cortical neurons after OGD (0.91 ± 0.032 vs. 0.57 ± 0.030, *p* < 0.05). U0126, an inhibitor of the Raf/MEK/ERK pathway, was used to mimic the role of ERK pathway in primary cortical neurons after the OGD treatment. After the treatment, the death rate of the Lv-Nnt-1 + U0126 group increased significantly when in comparison with that of the Lv-Nnt-1 group (4.50 ± 0.17 vs. 3.46 ± 0.25, *p* < 0.05) and the apoptosis rate of the Lv-Nnt-1 + U0126 group also elevated when compared with that of the Lv-Nnt-1 group (28.37 ± 0.50 vs. 18.20 ± 0.72%, *p* < 0.001). Altogether, these results indicate that Netrin-1 regulates the death rate and apoptosis of primary cortical neurons via the ERK pathway (Figures 4, 5).

### DCC Is Up-Regulated by Netrin-1 Expression

**Figure 6:**
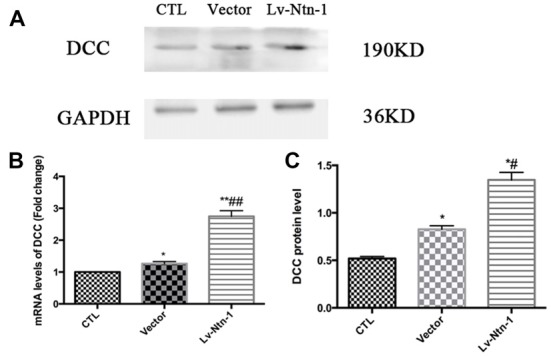
Deleted in colorectal cancer (DCC) was up-regulated by Netrin-1 expression. **(A)** The image of the detection of DCC by western blot. **(B)** Analysis of the DCC mRNA levels. Data are presented as mean ± SEM. *p < 0.05, as compared with the control group; ^#^*p* < 0.05, as compared with the vector group; **p < 0.01, as compared with the control group; ^##^*p* < 0.05, as compared with the vector group; *n* = 3. **(C)** Analysis of the DCC protein levels. Data are presented as mean ± SEM. *p < 0.05, as compared with the control group; ^#^*p* < 0.05, as compared with the vector group; Mann-Whitney U-test; *n* = 4.

**Figure 7:**
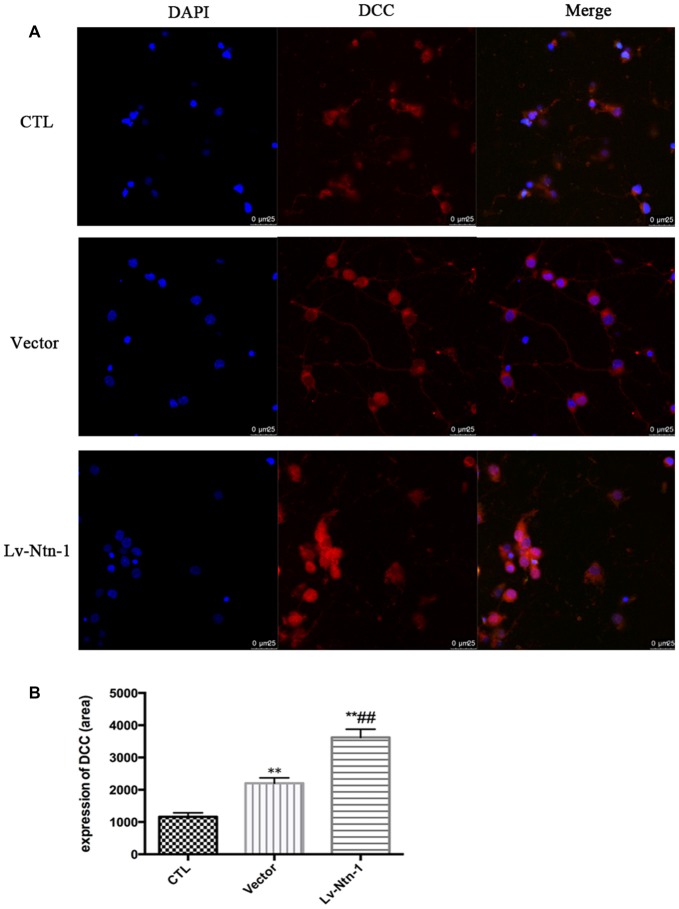
DCC was up-regulated by Netrin-1 expression. **(A)** The image of the detection of DCC by immunofluorescence. Scale bar = 25 μm. **(B)** Analysis of the DCC expression by calculating the DCC fluorescence area. Data are presented as mean ± SEM. **p < 0.01, as compared with the control group; ^##^*p* < 0.01, as compared with the vector group; One-way ANOVA (*F*_0.05(2,6)_ = 124.556); *p* < 0.001; *n* = 3.

Quantitative real-time PCR was used to measure the mRNA level of DCC. The analysis revealed that the mRNA level of DCC was significantly higher in the Lv-Nnt-1 group when compared with that of the vector group (2.75 ± 0.10 vs. 1.27 ± 0.04, *p* < 0.001). The protein expression of DCC was measured by western blot. As reported, the Lv-Nnt-1 group showed a much higher protein expression than the vector group (1.35 ± 0.04 vs. 0.83 ± 0.02, *p* < 0.05). To confirm the results of western blot, DCC were immunostained in different groups. A larger DCC fluorescence area was evident in the Lv-Nnt-1 group than in the vector and control groups (3620.96 ± 149.05 vs. 2200.65 ± 97.02 or 1159.57 ± 71.69, *p* < 0.01). As a membrane receptor, the DCC signal was located around the periphery of primary cortical neurons, and DCC signal was weak in control group and vector group (Figures 6, 7).

### Anti-DCC Reduces the Phosphorylation of ERK Signaling Pathway

**Figure 8:**
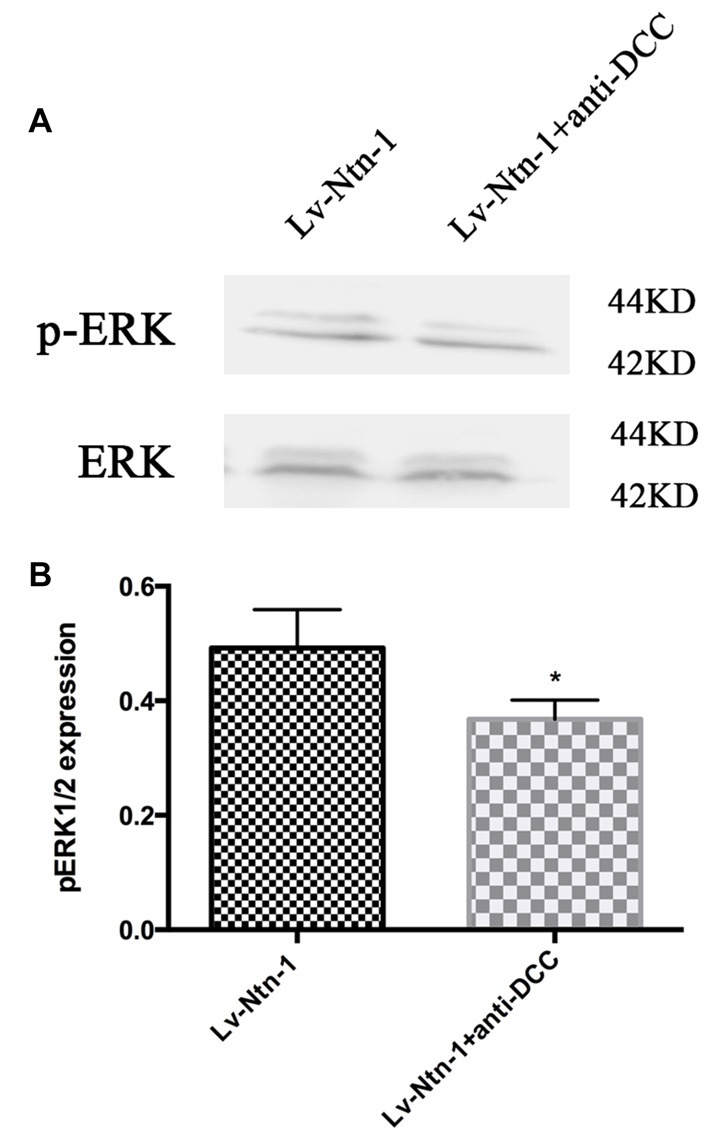
Anti-DCC reduced the phosphorylation of ERK signaling pathway. **(A)** The image of detection of ERK and phosphorylated ERK. **(B)** The analysis of pERK expression by western blot. Data are presented as mean ± SEM. *p < 0.05, as compared with the Lv-Ntn-1 group; Mann-Whitney U-test; *n* = 4.

As DCC and ERK are both involved in the anti-apoptotic effect of Netrin-1, we further investigated whether blocking DCC would affect the ERK signaling. The result of western blot revealed that anti-DCC treatment prevented the activation of ERK signal pathway (0.50 ± 0.06 for the Lv-Nnt-1 group vs. 0.37 ± 0.02 for Lv-Nnt-1 + anti-DCC group, *p* < 0.05), suggesting that in the Lv-Nnt-1 group, DCC may play a vital role in decreasing cell death and apoptosis (Figure 8).

### Netrin-1 Protects Primary Cortical Neurons by Reducing DNA Damage

**Figure 9:**
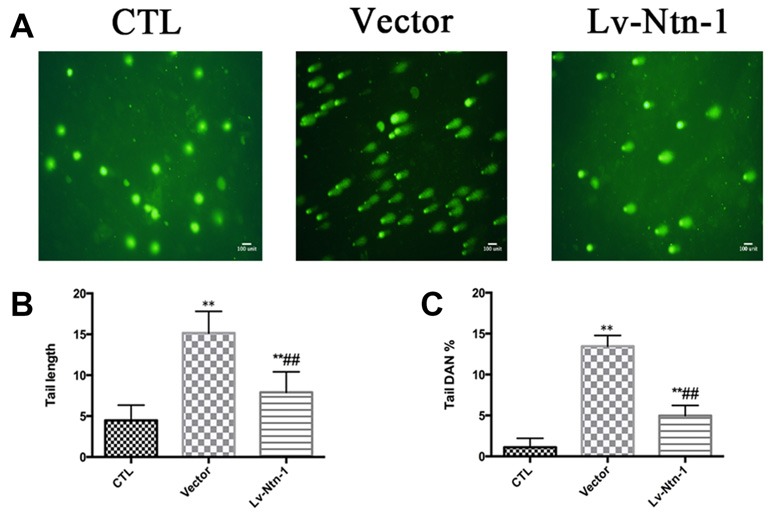
Netrin-1 protected primary cortical neurons by abolishing DNA damage. **(A)** The image of comet assay. **(B)** Analysis of the tail length of each group. Data are presented as mean ± SEM. **p < 0.01, as compared with the control group; ^##^*p* < 0.01, as compared with the vector group. **(C)** Analysis of the tail DNA (%) of each group. Data are presented as mean ± SEM. **p < 0.01, as compared with the control group; ^##^*p* < 0.01, as compared with the vector group; Mann-Whitney U-test; *n* = 3.

We used comet assay to measure DNA damage. Tail DNA and tail length were detected in different groups. The control group presented a normal nuclear matrix in the nucleus. However, the vector group has a higher level of DNA damage than the Lv-Nnt-1 group (Tail DNA: 13.46 ± 0.17 vs. 4.97 ± 0.18%, *p* < 0.01; Tail length: 15.16 ± 0.37 vs. 7.90 ± 0.35, *p* < 0.001; Figure 9).

## Discussion

In the present study, we speculated whether Netrin-1 could protect neurons from OGD-induced cell death and apoptosis with rat primary cortical neurons. We found that in addition to its role in neuronal migration, Netrin-1 also acted as a survival factor in the cultured primary cortical neurons that were subject to OGD treatment. Moreover, we found that with Netrin-1 overexpression, one of its receptor, DCC, was significantly up-regulated on the protein and mRNA levels, which indicates that Netrin-1 regulates the transcription of DCC. The ERK signaling pathway was involved in the protective effect of Netrin-1 and was activated by Netrin-1 overexpression, which was partly abolished by blocking the receptor DCC. Blocking the ERK signaling with the inhibitor U0126 reversed Netrin-1-induced anti-apoptosis. Netrin-1 protected primary cortical neurons by reducing DNA damage.

Netrin-1 is involved in the survival mechanisms of various human cancer cell lines before it is silenced by siRNA (Delloye-Bourgeois et al., 2009a; Paradisi et al., 2013). Netrin-1 siRNA is associated with a potentiation of cancer cell death in the Doxorubicin treatment, which appears to be p53-dependent (Paradisi et al., 2013). Mazelin et al. (2004) also showed that inhibition of cell death by enforced expression of Netrin-1 in mouse gastrointestinal tract leads to the spontaneous formation of hyperplastic and neoplastic lesions.

In the research of cerebral ischemia, *in vivo*, Netrin-1 was up-regulated after middle cerebral artery occlusion (Liu et al., 2011). The current study employed an *in vitro* design and found that Netrin-1 overexpression markedly inhibited neuronal apoptosis when the cultured neurons were subject to OGD condition.

In the model of ischemia/reperfusion-induced myocardial infarction, with Netrin-1 treatment, Netrin-1 receptors were detected, in which the mRNA and protein level of DCC were abundantly elevated (Zhang and Cai, 2010), which is consistent with our research.

In the current study, we found that the ERK signaling pathway was involved in the Netrin-1-induced protection of neurons. The ERK signaling pathway was involved in different pathological conditions (Li et al., 2012; Tan et al., 2013; Wu et al., 2016). Its activation plays an important role in the proliferation of neural progenitor after cerebral stroke (Kalluri et al., 2007). With the presence of U0126, an inhibitor of MEK1/2, the cultured neural progenitors were decreased after OGD compared with the one without U0126. In the research of Chang et al. (2017), the neurite outgrowth was promoted by ARDD-activated ERK phosphorylation, which could be reversed by PD98059, an inhibitor of the ERK pathway. In Sun et al.’s (2017) research, the neuronal apoptosis was significantly affected by NGF/HO-1-activated MEK/ERK pathway after the cultured primary cortical neurons were exposed to OGD.

In our study, Netrin-1 overexpression also activated the ERK signaling pathway, which was partly blocked by blocking the receptor DCC. Blocking the ERK signaling with the inhibitor U0126 reversed the effect of netrin-1-induced anti-apoptosis, which is consistent with the findings reported by the studies mentioned above.

In the research of Zhang and Cai (2010), Netrin-1 protected the heart from myocardial infarction via the activation of the ERK signaling pathway, which was abolished by U0126, indicating that Netrin-1 plays a role in myocardial infarction via the ERK signaling pathway. In his study, the ERK signaling inhibitor reduced DCC expression, indicating a DCC/ERK1/2/eNOSs1177/NO/DCC feed-forward mechanism.

In our study, we found that the activity of ERK signaling pathway was associated with DCC when Netrin-1 was overexpressed. In the analysis, the ERK pathway was inhibited by blocking DCC, but the exact mechanism has not been elucidated yet. In the research of Ma et al. (2010), there are three conserved sequence motifs, namely, P1, P2 and P3 in cytoplasmic of DCC. The P1 domain of DCC was bound to ERK2 directly, which is in agreement with our result that Netrin-1/DCC complex activates the ERK signaling pathway.

We also found that not only DCC, but the other two receptors of Netrin-1, Unc5B and Unc5C were also increased in the mRNA level after OGD. It remains unclear if Unc5B and Unc5C also participate in the activation of the ERK signaling pathway. The ERK signaling pathway was reduced in MiaPaCa II cells with Netrin-1 overexpression (An et al., 2016), which is not consistent with our study. However, in their research, only Unc5B was dramatically increased among the receptors of Netrin-1 and DCC did not increase obviously in MiaPaCa II cells. We assume that the ERK signaling pathway is activated by DCC instead of Unc5B. There is no evidence that Unc5C is involved in the mediation of the ERK signaling pathway.

In our study, Netrin-1 reduced DNA damage. We speculate that this effect is achieved through the activation of the ERK signaling pathway. As well documented in existing literature, DNA damage can induce cell cycle arrest or apoptosis while the ERK signaling pathway can modulate the cell cycle arrest or apoptosis induced by DNA damage (Lin et al., 2013; Chitikova et al., 2014; Yeh et al., 2014; Liao et al., 2015). A recent study shows that the knockdown of either ERK1 or ERK2 can abolish the Etoposide-induced G2/M arrest (Wei et al., 2010). Another research indicates that the ERK pathway affects the checkpoint activation in DNA damage response, in which the activation of ERK pathway promotes DNA-damage-induced G1/S arrest (Tentner et al., 2012). In addition, the inhibitor of ERK has been reported to reduce the activation of ATM and ATR, two DNA damage response-related kinases (Golding et al., 2007). With the DNA damage, the Double stranded DNA breaks (DSBs) activate DSB repair by homologous recombination (HR). The ERK inhibitor reduces the HR in U87 cells, which demonstrates that the ERK signaling pathway facilitates HR (Wei et al., 2010).

## Conclusion

In cultured primary cortical neurons, Netrin-1 overexpression prevents neurons from apoptosis after OGD via the DCC/ERK pathway, in which Netrin-1 mediates the level and transcription of DCC. In addition, Netrin-1 also reduces the DNA damage of neurons.

## Author Contributions

JC drafted the main manuscript and performed the main experiments. HD participated in flow cytometry experiments. NL conceived and designed the experiments. YZ revised the manuscript. PL helped the culture of cortical neurons, western blotting and immunofluorescence staining experiments. JC and QL took part in qPCR experiments. RC was responsible for analyzing the data. All authors read and approved the final manuscript.

## Conflict of Interest Statement

The authors declare that the research was conducted in the absence of any commercial or financial relationships that could be construed as a potential conflict of interest.
